# High-Yield Lignocellulosic Fibers from Date Palm Biomass as Reinforcement in Polypropylene Composites: Effect of Fiber Treatment on Composite Properties

**DOI:** 10.3390/polym12061423

**Published:** 2020-06-26

**Authors:** Chihaoui Belgacem, Quim Tarres, Francesc Xavier Espinach, Pere Mutjé, Sami Boufi, Marc Delgado-Aguilar

**Affiliations:** 1 Faculty of Science, University of Sfax, LMSE, Sfax BP 802-3018, Tunisia; chihaoui.belgacem@gmail.com (C.B.); Sami.Boufi@fss.rnu.tn (S.B.); 2 LEPAMAP Research Group, Department of Chemical Engineering, University of Girona, 17003 Girona, Spain; pere.mutje@udg.edu; 3 Càtedra de Processos Industrials Sostenibles, University of Girona, 17003 Girona, Spain; 4 PRODIS Research Group, Department of Chemical Engineering, University of Girona, 17003 Girona, Spain; francisco.espinach@udg.edu

**Keywords:** date palm waste, composites, interface, enzymatic treatment, tensile properties

## Abstract

In this work, date palm waste (DPW) stemming from the annual pruning of date palm was used as reinforcing filler in polypropylene (PP) matrix at 40% *w*/*w*. Three pre-treatment routes were performed for the DPW, namely (i) defibration, (ii) soft alkali treatment, and (iii) enzymatic treatment, to obtain date palm fibers (DPF) and to investigate the effect of each process on their chemical composition, which will ultimately affect the mechanical properties of the resulting composites. The enzymatic and alkali treatment, combined with maleated polypropylene (MAPP) as a coupling agent, resulted in a composite with higher strength and stiffness than the neat PP. The differences in the reinforcing effect were explained by the change in the morphology of DPF and their chemical surface composition according to the selected treatment of DPW. Enzymatic treatment maximized the tensile strength of the compound as a consequence of an improvement in the interfacial shear strength and the intrinsic resistance of the fibers.

## 1. Introduction

Natural fiber-reinforced composites are a form of composite produced by means of blending woody and/or non-woody biomass fibers with a matrix, usually polyethylene or polypropylene, usually at ratios of between 20 and 60 wt %. This type of material, which combines the qualities of fibers and the ease of workability of plastic, has been increasingly developed over the last twenty years, and their application areas have expanded in outdoor applications and construction-related materials. Examples of applications include outdoor and indoor wood-like furniture, decking and boardwalks, flooring, window and door profiles proof, outdoor building trims, interface elements in construction and automotive interior parts, among others [1].

The use of such composites offers numerous advantages, such as (i) the substitution of a large fraction of petrochemical materials with bio-based materials deriving from biomass, (ii) the reduction of landfilled waste, (iii) high durability with high resistance to fading, staining and scratching, (iv) low maintenance requirements with good dimensional stability, (v) aesthetic characteristics of wood, and (vi) good thermal and acoustic insulating properties [2,3].

Although woody and non-woody fibers remain the main source for the production of natural fiber-reinforced composites, in practice, any resource of lignocellulosic fibers could also be used for this purpose, such as agricultural crop residue, side streams from agricultural and industrial activities or annual plants. Date palm, a member of the palm tree family (Phoenix dactylifera) is extensively cultivated in North and Middle Africa for date fruit exploitation. Apart from date fruit, which is highly nutritional, date trees also produce large quantities of agricultural waste and side streams stemming from the pruning of date palm, which roughly accounts for 50 kg per year [4]. This pruning is needed to remove old, dead or broken leaves and improves the quality of the fruit.

Although the abovementioned wastes offer a potential alternative to virgin resources, apart from increasing the added value of such residues, the processing of such resources must be conducted under the premise of generating the minimum waste from waste. In this sense, mechanical fibrillation, also known as mechanical pulping, offers an adequate balance between fiber quality (moderate slenderness ratios) and yield (higher than 95%). However, mechanical pulping critically damages the fibers and treatments to soften lignin and other compounds in lignocellulosic resources must be conducted. Thus, alkali and enzymatic treatments are interesting for achieving adequate morphology and surface chemical compositions, contributing to the fiber-matrix adhesion and promoting stress transfer from matrix to reinforcement.

Tunisia is one of the leading date palm producers and is the first exporting country (in value) in the world, with an average annual production of about 120 thousand tons, more than four million date palm trees, and about 250 cultivars grown extending over about 32,000 hectares of oases [5,6]. This agricultural activity generates a large amount of date palm rachis and leaf waste consisting mainly of cellulose, hemicelluloses and lignin, and most of this is left to rot or open-air burned in fields, releasing harmful oxides that contribute to an increase in air pollution. This abundant biomass can be considered a source of lingocellulosic fiber with potential applications in composite materials, particleboards and insulating panels, among other things. An interesting review compiling current literature related to date palm fibers, their extraction techniques, characteristics, and applications, was recently published by [7]. For instance, date palm tree by-products have been used as a starting material to produce particleboard composites by hot pressing without any pretreatment or addition of synthetic binders as self-bonded materials [8,9]. It was shown that the mechanical resistance and internal bond strength were dependent on the chemical composition of the original raw material, particularly the lignin and extractive content. The use of date palm residue as reinforcement or filler in polymer is another potential application arousing a great deal of interest [10]. In addition, a wide range of polymer matrices, including polyolefin (PE, PP) [8,11,12], thermosets [13], polyurethane [14] and biodegradable polymers [15,16], have been explored with broad results in terms of the reinforcing efficiency of the date palm fibers. For instance, polyethylene composites based on date palm flour with contents ranging between 40 and 75 wt % were investigated by Mirmehdi et al. (2014) [17] using twin screw extrusion and hot pressing as a processing route and maleic anhydride-modified polyethylene (MAPE) as a coupling agent. However, no reinforcing effect was found, while a huge decrease in the strength (flexural and tensile) with respect to the neat matrix was observed. In contrast, previous studies have shown that the incorporation of mechanical fibers to PP, together with the addition of coupling agent (MAPP), provides significant increases in mechanical properties [18,19].

The use of date palm leaf fiber as filler in poly (ethylene terephthalate) (PETr) matrix with content up to 15 wt % was reported by Dehghani et al. (2013) [20]. Using maleic anhydride-grafted poly (styrene-ethylene/butyl-dienestyrene) (SEBS-g-MA), an impact modifier bearing reactive group, a beneficial effect on the tensile and flexural strength and flexural was noted along with an increase in the onset crystallization temperature and crystallinity degree. In another study, bleached date palm fibers after soda treatment were used to produce composites based on PP and low-density polyethylene LDPE matrix [21]. However, while the inclusion of the fibers provided a significant increase on the stiffness, only a marginal effect on the tensile strength was observed, meaning that the fibers behave as a filler rather than a reinforcement. Regarding thermosets, in epoxy-based composites, NaOH-treated date palm fibers at a concentration of 6 wt % were found to be the optimal treatment to maintain high interfacial adhesion and strength [22].

In most of the literature, the date palm residue is often used in the form of ground powder with optional sieving treatment to remove coarse particle and reduce particle size distribution. Another widely adopted strategy consists on performing an alkali treatment to partially remove lignin and promote the individualization of fibers. These different processing strategies will strongly affect the fiber morphology (length and diameter) and surface properties, making it hard to compare how the fibers’ inclusion in a polymer matrix affects the mechanical property of the composite, its water sensitivity and fiber dispersion within the polymer matrix. Actually, in short fiber composites, the reinforcing effect of fibers is subjected to the efficiency of stress transfer from the matrix to fibers through the interfacial area. This effect is known to be strongly dependent on several parameters, such as the aspect ratio of fibers, the interfacial fiber–matrix adhesion or interfacial shear strength factor (IFSS), fiber dispersion, fiber orientation within the matrix, and the intrinsic mechanical properties of fibers (tensile strength and modulus). A high degree of adhesion between fibers and matrix is desired in order to obtain the best mechanical properties, reducing the effect of water and the porosity.

For environmental reasons, researchers have rushed to find new solutions to reduce the use of chemicals in fiber treatment; in this regard, it has been demonstrated that the use of enzymes is an alternative way [23]. Enzymatic action is clearly sustainable due to its selective character and high yields. Nowadays, xylanases are the major commercial proportion of hemicellulase [24]; however, due to their advantages of prebleaching, such as higher pulp brightness [25], these enzymes have attracted increasing attention in the pulp and paper industry [26], which has pushed enzyme producers to develop more new factories [25] in order to produce sufficiently high quantity. For economic and environmental reasons, [26] studied the effect of xylanase pretreatment of rice straw soda and neutral sulfite pulps to produce nanofibers. They concluded that with xylanase treatment, they could successfully isolate nanofibers with increasing mechanical network properties. In another study, [27] studied xylanase pretreatment for isolation of microfibrillated cellulose (MFC) from date palm fruit stalks. Xylanases pretreatment date palm resulted in MFC with higher tensile strength properties and physical properties such as density, lower air permeability and lower water absorption compared to the MFC isolated from untreated pulp.

Given the wide discrepancy on the reinforcing capacity of date palm fibers as polymer matrix reinforcement, it is imperative to study how the biomass preparation strategy affects their reinforcing potential. Based on this assessment, three preparation strategies of the date palm residue were adopted, namely mechanical disintegration, alkali treatment and enzymatic treatment (combination of xylanase and pectinases). These treatments should make it possible to obtain high yield lignocellulosic fibers from date palm biomass. Composites based on PP matrix with 40% *w*/*w* fiber content were processed in the presence of 5 wt % MAPP, and their mechanical and mircomechanical properties were investigated. Understanding how the treatment route of DPW residue is likely to affect their reinforcing potential would contribute to better exploit this important agricultural side streams in composite manufacturing with higher added value.

## 2. Materials and Methods

### 2.1. Materials

Date palm waste (DPW) was obtained from different parts of the date palm tree, rachis, leaflet and leaf, resulting from the annual pruning of date palm tree in the region of Gabes (Tunisia).

The selected polymer matrix was polypropylene (PP) Isplen PP090 G2M (Repsol Química, S.A., Tarragona, Spain). According to the supplier, this PP exhibited a melt flow index of 35 g/10 min and a density of 905 kg/m^3^. Maleic anhydride polypropylene (MAPP) Epolene G-3015 from Eastman Chemical Products (San Roque, Spain) was used as a coupling agent between the fibers and PP. According to the supplier, the Epolene G-3015 exhibited a molecular weight of 47,000 g/mol with an acid number of 15 mg KOH/g.

Xylanases (Panzea^®^) and pectinases (Pectinex^®^ XXL) were provided by Novozymes (Bagsvard, Denmark).

### 2.2. Methods

**Figure 1:**
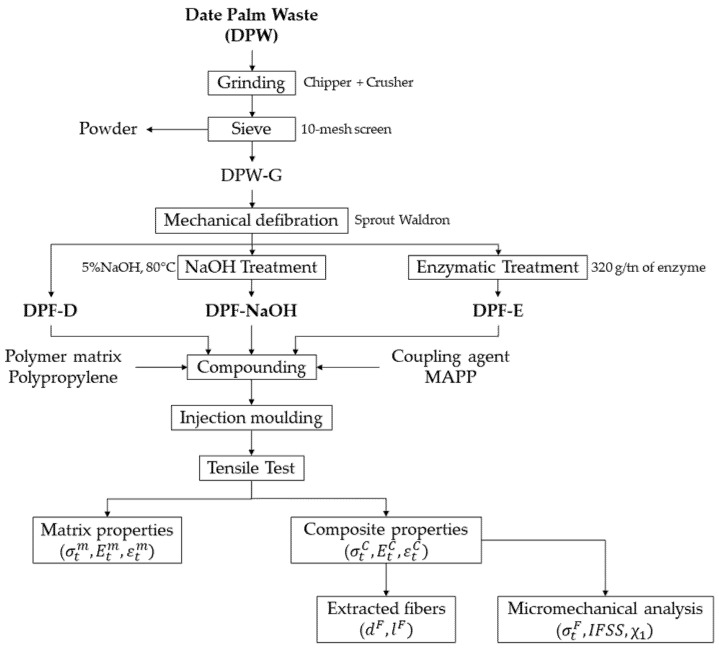
Flow chart of the present study.

Figure 1 shows the general workflow of the present study, from the preparation of the different date palm fibers (DPF) from the date palm waste (DPW) to the production and characterization of the composite materials.

#### 2.2.1. Preparation of the DPF

First of all, the DPW was air dried and ground with a chipper into the form of coarse particles 10–30 mm in length. The resulting ground DPW (DPW-G) was passed through a Sprout–Waldron defibrator until a constant diameter was reached (DPF-D). Then, the DPF-D was submitted to two different treatments. On the one hand, the alkali treatment was performed by means of soaking the DPW in a 5% NaOH solution at 80 °C for two hours. Then, the fibers were washed thoroughly with deionized water to remove the excess of NaOH until a neutral pH was reached. The resulting fibers were labelled as DPF-NaOH. On the other, an enzymatic treatment based on xylanase and pectinase enzymes was performed. The enzyme solution was first dissolved in their corresponding buffer (phosphate buffer at pH 7 for xylanase and acetate buffer pH 4.8 for pectinase). Then, 50 g of DPW was mixed with 1 L of the enzyme solution at a concentration of 100 U/g of DPW for xylanase and 3000 U/g DPW for pectinase, and the suspension was kept under mechanical stirring at 50 °C for 24 h. The fibers were then recovered by filtration followed by washing three times with water until a nearly neutral pH was obtained. The fibers were dried at room temperature for two days and at 50 °C for 24 h to completely remove the water.

#### 2.2.2. Fiber Characterization

The chemical composition of the fibers after each treatment was determined in terms of extractives, ash, lignin and holocellulose content. Initially fibers were dried at 105 °C for 24 h; TAPPI T264 cm-97. Extractives content was determined by ethanol–benzene extraction following the standard TAPPI T204 cm-97. Ash content was measured by gravimetric analysis according to TAPPI standard T211. Holocellulose content was calculated by difference from 100% [28,29]. Kappa number was measured using a simple methodology to estimate lignin content following the TAPPI T236 cm-85 standard.

Fibers were recovered from the composite materials using a Soxhlet extraction. Decalin was used as a plastic solvent. The fibers obtained were cleaned several times with distillated water and their morphology was characterized using a MorFI compact analyzer (TechPap, Gières, France). Morphological analysis reported the averages of fiber length distribution, length and diameter as the main parameters.

#### 2.2.3. Preparation of the Composite Materials

Composites were prepared in an internal mixer Brabender Plastograph^TM^ (Duisburg, Germany), controlled by WINMIX software (Brabender, Duisburg, Germany), which, at the same time, displayed mechanical information about the mixing process. The mixing chamber, equipped with two screws counter-rotating at 80 rpm and preheated to 190 °C, had a loading capacity of 50 cm^3^. First of all, PP was introduced into the mixing chamber and, once the torque remained constant, MAPP was introduced at a ratio of 5% with respect to the amount of fiber that was later introduced. Then, fibers (either DPF-D, DPF-NaOH or DPF-E) were introduced into the mixing chamber and kept until a constant torque was achieved again. Once the composite material was discharged from the mixing equipment, it was passed through a blade mill equipped with a screen of 10 mm at the bottom. The granules were then processed by injection molding using Aurgburg 220 M 350-90U equipment (Lossburg, Germany) equipped with a mold for standard specimens, according to the ASTM D3641 standard for tensile characterization. The machine was equipped with five heating zones, whose temperatures were set at 175, 180, 185, 185 and 190 °C, respectively, and the pressure ranged from 300 to 600 bar. The specimens were then conditioned according to the ASTM D618 standard before testing (23 °C and 50% of relative humidity for 48 h). The amount of DPF was set at 40 wt %.

#### 2.2.4. Composites Characterization

The obtained materials were tested according to the ISO 527-1:2012 standard using an Instron^TM^ 1122 universal testing machine (supplied by Metrotec, S.A., Barcelona, Spain) fitted with a 5 kN load cell. The gap between clamps was set at 115 mm with a cross-head testing velocity of 5 mm/min. The Young’s modulus was determined by using an extensometer, and the test was stopped before breaking the sample. A minimum of five specimens were tested per test.

Scanning Electron Microscopy (SEM) observations were performed with the aim of analyzing the microstructure of the fracture surface of the test specimens. This observation was carried out with a FESEM HITACHI S4100 (HITACHI, Mannheim, Germany) and Everhart-Thornley secondary electron detector (HITACHI, Mannheim, Germany). The surface to be observed was coated with carbon to avoid the sample charging effect due to the electron beam.

## 3. Results and Discussion

### 3.1. Tensile Properties of Date Palm Fibers—PP Composites

As explained in the previous section, 40 wt % reinforced composites were prepared from each type of DPF (DPF-D, DPF-NaOH and DPF-E) using 5 wt % of MAPP as a coupling agent. The results of the tensile tests are summarized in Table 1, where $V^{F}$ is the volume fraction of the fibers in the composite, $\sigma_{t}^{C}$ is the tensile strength of the composite, $E_{t}^{C}$ is the Young’s modulus, $\varepsilon_{t}^{C}$ is the strain at break, $\sigma_{t}^{m^{*}}$ is the matrix contribution to the tensile strength of the composite, and $d^{F}$ and $l_{w,w}^{F}$ are the diameter and weight-weighted length of the extracted fibers.

**Figure 2:**
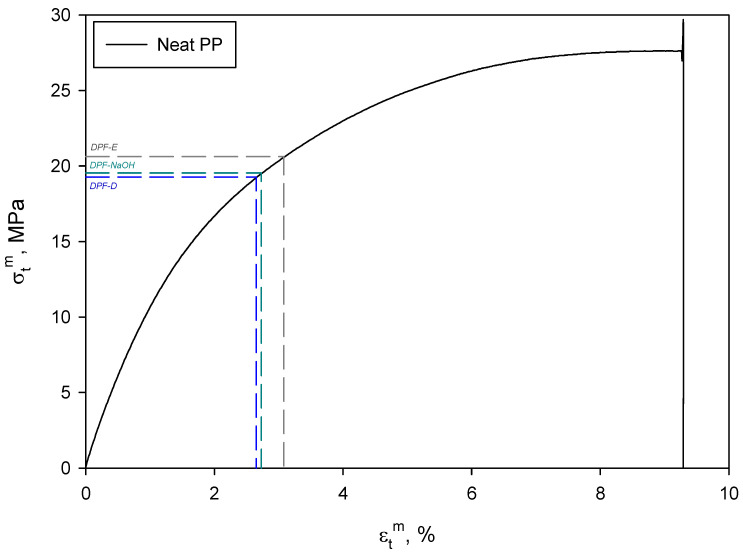
Determination of matrix contribution for the different composite materials.

As can be seen, the Young’s modulus significantly increased when DPF were incorporated. Concretely, the enhancement of this property corresponded to 161, 190 and 205% for DPF-D, DPF-NaOH and DPF-E, respectively. As has been previously reported, Young’s modulus is independent from the interface between the matrix and the reinforcement and is indicative of the stiffness of the material, as well as the reinforcement distribution within the matrix [30]. Thus, the increase in Young’s modulus mainly comes from the fibers having a higher modulus than the matrix. Considering that the different fibers imparted different enhancements on the Young’s modulus, the intrinsic modulus of the fibers might also be different. In fact, according to the results, it is expected that DPF-E would present a higher intrinsic modulus than DPF-D or DPF-NaOH. In principle, an increase in the Young’s modulus of a material will lead to a decrease in its strain at break [31,32]. However, the DPF-E exhibited the highest Young’s modulus, but also the highest strain at break among the composites, indicating that fiber morphology also plays a key role in this property [33]. In fact, DPF-E exhibited the lowest diameter and length, but the highest slenderness or aspect ratio. This higher deformation at break indicates a greater contribution of the matrix in such materials, as can be observed in Figure 2.

With respect to tensile strength, it was found that the three types of DPF imparted a significant enhancement, achieving an increase of almost 90% with respect to the strength of the PP matrix in the case of DPF-E. Considering that DPF-D underwent a similar process to those commercially available mechanical pulps, the reinforcing effect was slightly lower. In fact, López et al. (2012) [19] found that the incorporation of 40 wt % of stone groundwood pulp from pine improved the tensile strength of PP by 86%, reaching an absolute value of 51.4 MPa. This effect was also found for DPF-NaOH, where the incorporation of alkali-treated fibers into PP was reported to impart a greater enhancement. These differences may be due to different reasons, including differences in the nature of the fibers, their morphology and, in fact, that the amount of MAPP in this study was slightly lower.

Overall, according to Thomason (2002) [34], the tensile strength of a composite material strongly depends on the matrix properties, the intrinsic properties of the reinforcement, the interfacial shear strength (IFSS), the nature of the reinforcement, the orientation of the reinforcement with respect to the stress direction, the aspect or slenderness ratio (length to diameter) and the homogeneity of the reinforcement distribution within the matrix. In fact, the observed tensile strengths of the different composite materials follow a similar tendency than the aspect ratio, although the differences are almost insignificant. Therefore, the properties of composite materials can be estimated from the modified rule of mixtures (Equation (1)).

(1) $$\sigma_{t}^{C}=(\chi_{1}\cdot \chi_{2})\cdot \sigma_{t}^{F}\cdot V^{F}+\sigma_{t}^{m^{*}}\cdot \left(1-V^{F}\right)$$

where the tensile strength of the composite ($\sigma_{t}^{C}$) is the sum of the matrix contribution ($\sigma_{t}^{m^{*}}\cdot (1-V^{F}$)), the reinforcement contribution ($(\chi_{1}\cdot \chi_{2})\cdot \sigma_{t}^{F}\cdot V^{F}$), the intrinsic tensile strength of the fiber ($\sigma_{t}^{F}$), and the volume fraction of the reinforcement ($V^{F}$), multiplied by the coupling factor ($f_{c}$). The coupling factor includes both the orientation factor ($\chi_{1}$) and the length and interface factor ($\chi_{2}$), as reported by Sanadi et al. (1994) [35].

Comparing the obtained results, it becomes apparent that DPF-D exhibited the lowest mechanical performance, exhibiting 5 and 11% less strength than DPF-NaOH and DPF-E, respectively. Although the amount of reinforcement, by mass, was the same in the three different composites, it must be taken into account that the volume fraction was not. The density changes of the fibers took place to a different extent depending on the selected treatment. However, the differences observed in the volume fraction may not have had a direct influence on the resulting tensile strength, since DPF-E exhibited the lowest V^F^, while exhibiting the highest strength. The orientation and distribution of the reinforcement within the matrix may not be the parameter generating the differences, since the mold geometry during processing was the same, as well as the processing conditions (mainly flow, pressure, temperature and cooling rate). In addition, MAPP was incorporated into the composites at the same ratio, regardless of the type of fiber, and always as a function of the mass fraction of reinforcement. Furthermore, the slenderness ratio changed with the different treatments, with the highest value being obtained in the case of DPF-E, and the lowest in the case of DPF-D. The aspect ratio could even be improved by using a kinetic mixer for compounding instead of the internal mixer selected for the present work. This improvement to the aspect ratio, which is mainly due to the lesser effect on fiber shortening, could enhance the tensile strength of the resulting composite materials [2]. However, the dispersion of the reinforcement when kinetic mixers are used is usually worse than in the case of internal mixers.

For all of the above, it is apparent that the different behavior of the obtained DPF may be due to the differences in their chemical compositions, which, in fact, have a direct effect on the IFSS. In fact, this IFSS mainly depends on the type of bonds that are generated between the matrix and the reinforcement and the number of bonds per volume unit [3]. In this sense, the surface chemical composition of the fibers is determinant in the extent of the reaction between the hydroxyl groups from the surface of the fibers and the maleic anhydride rings of MAPP [36]. Table 2 shows the chemical composition of DPF-D, DPF-NaOH and DPF-E.

**Figure 3:**
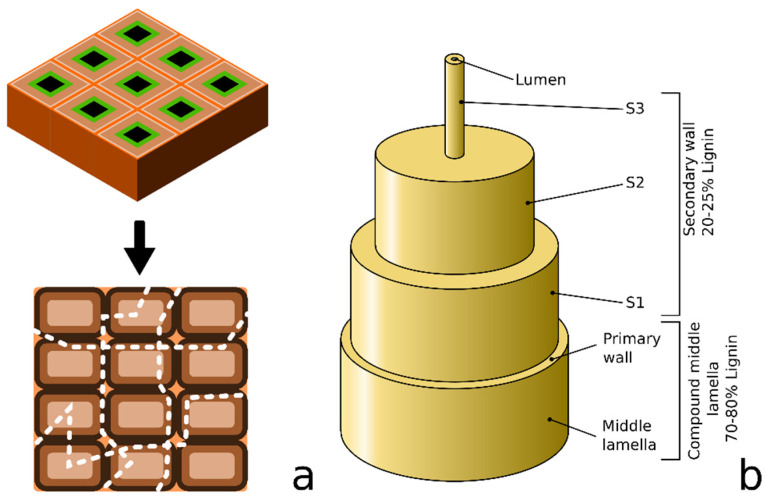
(**a**) Mechanism through which fibers are separated during mechanical fibrillation and (**b**) Scheme of natural fiber structure.

The obtained results show that the three types of DPF exhibited a high lignin content, especially DPF-D, which was only mechanically treated. In fact, the mechanical treatment does not usually modify the chemical composition of the raw material, other than removing small amounts of extractives that are soluble in cold water, and a part of the ashes [37]. Figure 3a proposes a mechanism through which fibers are separated during mechanical fibrillation if no other treatments have previously been performed on the fibers. In fact, if lignin has not been previously softened, the shear stress caused by mechanical fibrillation will randomly break the structure of the material, leading to heterogeneous surfaces and morphologies [38]. Figure 3b proposes a model of a single lignocellulosic fiber, where the different layers can be easily identified. At the outer part of the fiber (compound middle lamella), the model proposes that between 70% and 80% of the lignin is present. Alkali treatments have been previously reported to be effective for lignin removal, and this process is widely used in papermaking. In this sense, the combination of the mechanical action and the alkali treatment conducted in this work may contribute to the removal of lignin along with better separation of the fibers. Thus, the 2% decrease in lignin content is understandable, increasing the relative content of holocellulose.

**Figure 4:**
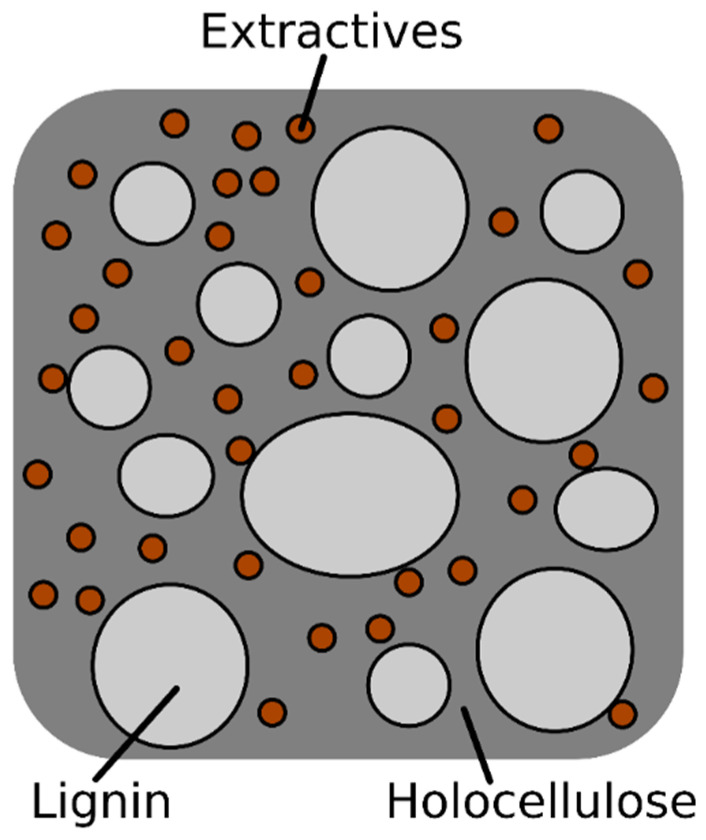
Scheme of chemical surface composition of natural fibers (adapted from Böras and Gatenholm (1999) [41]).

On the other hand, the enzymatic treatment carried out with xylanases and pectinases enables a larger removal of extractives, ashes and lignin. It is well known that part of the extractives contained in lignocellulosic fibers consist largely of pectin, among other things. Therefore, the action of the pectinases promotes the reduction of the extractive content of the fibers, reducing this content from 2.59 to 0.72% [39,40]. Furthermore, xylanases are usually used for hemicellulose degradation (xylans), while they are not oriented towards lignin or extractives removal [26]. However, the model proposed by Böras and Gatenholm (1999) [41], an adapted version of which can be observed in Figure 4, proposes that part of the lignin is bonded to the surface of the carbohydrates. The degradation of some hemicellulose may contribute to the release of the attached lignin with the black liquor derived from the enzymatic process. This phenomenon leads to a decrease in lignin content from 26.5 to 19.86%, as reported by De Prez et al. (2019) [40]. At the same time, the action of the pectinases and xylanases promotes the elimination of part of the ashes, which reached values below 2% in the case of DPF-E. The enzymatic treatment led to the DPF with the highest carbohydrate content (cellulose and hemicellulose), with 77.45% holocellulose. However, the ratio of hemicellulose to holocellulose may be affected. Cellulose content is the predominant component, which would explain a higher intrinsic properties of the fibers, as will be discussed later. The reduction of the lignin content was corroborated by the reduction in the Kappa number of the fibers.

**Figure 5:**
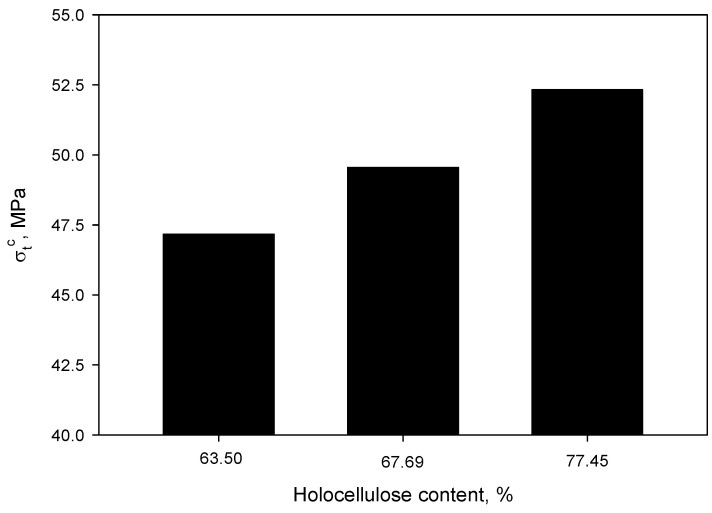
Evolution of tensile strength of the 40 wt % reinforced composites as a function of the holocellulose content in the reinforcement.

According to the proposed model, lignin and extractives cover the surface of carbohydrates, indicating that the higher the lignin content is, the less accessibility the hydroxyl groups from cellulose and hemicellulose have to bond to MAPP during the compounding process. In fact, analyzing the evolution of the tensile strength of the 40 wt % reinforced composites as a function of the holocellulose content of the fibers (Figure 5), it is apparent that the removal of lignin plays a key role in the mechanical performance of the composites.

Thus, the incorporation of fibers with higher holocellulose content leads to stronger composites. In fact, this confirms the interaction mechanism proposed above, where hydroxyl groups were assumed to play an important role in the interface between MAPP and DPF. Considering the proposed chemical surface composition and distribution (Figure 4), it is apparent that the removal of lignin may contribute to the generation of free hydroxyl groups at the surface of the fibers, promoting its interaction with the selected coupling agent.

**Figure 6:**
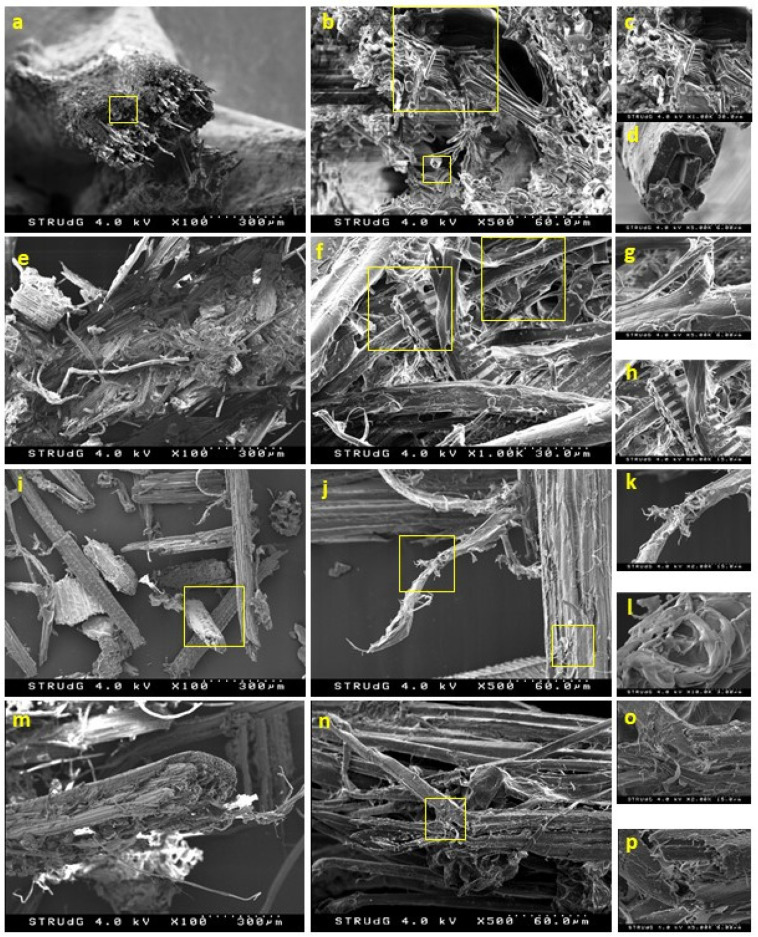
SEM micrograph showing the morphology of DPW (**a**,**b**,**c**,**d**), DPF-D (**e**,**f**,**g**,**h**), DPF-NaOH (**i**,**j**,**k**,**l**) and DPF-E (**m**,**n**,**o**,**p**) at different magnifications.

The morphology of the reinforcement, another parameter that plays an important role in the mechanical performance of the composites, was analyzed by means of SEM observation. The morphology of the raw material, prior to any of the selected treatments, was also analyzed for the purpose of comparison (Figure 6).

With respect to DPW, different fragments can be observed, namely xylem, vessel and fiber cells. These micro-structural elements have specific functions within the plants, such as ensuring the long-distance transport of water and mineral nutrients throughout the plant (vessels) or providing dimensional stability and mechanical strength (fiber cells). Fibers, in the form of bundles, bind together and are structured in parallel, exhibiting diameters of 10–20 µm, with a lumen of 5–10 µm and cell walls of about 2–3 µm. The geometry of fibers depends both on the location at and the period in which they were formed (thinner during the summer and thicker during the winter) [42,43]. Apart from the biomass itself, mineral residue at the surface can be observed.

Under the effect of mechanical grinding, DPW was merely broken into smaller particles without any separation of the fibers. After mechanical fibrillation (DPF-D), long fibrillary bundles were obtained without notable cutting action. This effect is presumably the result of the high compression and shearing forces exerted during passage through the discs, favoring the breakdown of DPW in their longitudinal direction. The alkali treatment further promoted the separation of the bundles, leading to better defined fibers, as in the case of the enzymatic treatment.

Morphological changes after the alkali treatment were to be expected, as it is well known that NaOH partially dissolves lignin, although it was performed at a low temperature and concentration for reasons of economy. However, the soft conditions of the alkali treatment limited the individualization of the fibers, and better results could be obtained under more stringent process conditions.

On the other hand, the cutting action and degradation produced by the enzymes promoted the release of the fibrils. The use of enzymes to obtain cellulose nanofibers has been widely reported [44,45,46]. In this sense, the combination of different enzymes, pectinases and xylanases promotes a greater separation of fibrillar elements.

### 3.2. Micromechanical Study

A micromechanical analysis was carried out in order to corroborate the role of lignin and morphology in the interface of the composites. As mentioned above, the properties of composite materials can be estimated based on the rule of mixtures (Equation (1)). However, in this equation, there are two unknown parameters: the intrinsic strength of the fibers and the coupling factor.

For this reason, a first approach to evaluating the micromechanics of composite materials can be carried out by calculating the fiber tensile strength factor (FTSF), expressed as: $FTSF=f_{c}\cdot \sigma_{t}^{F}$. Thus, if Equation (1) is applied, it is possible to obtain a factor of 112.8 for the composite with DPW-D, compared to 122.33 for the composite with DPF-NaOH fibers. Previous studies have reported similar behaviors for mechanical wood pulp fibers. The FTSF increased from 110.9 to 128.8 when they were treated with sodium hydroxide [2]. The tensile strength factor of the fibers reached its maximum value of 136.5 for the enzyme-treated fibers. However, further analysis of the micromechanics is required in order to determine whether this is due to greater intrinsic properties ($\sigma_{t}^{F}$) or a better interface ($f_{c}$).

Subsequently, a micromechanical analysis of the fiber–matrix interface was performed using the modified rule of mixtures of Kelly and Tyson (Equation (2)) and the solution proposed by Bowyer and Bader [47,48].

(2) $$\sigma_{t}^{C}=\chi_{1}\cdot \left(\overset{i=0}{\underset{Lc}{\sum }}\left[\frac{\tau \cdot l_{i}^{F}\cdot V_{i}^{F}}{d^{F}}\right]+\overset{j=Lc}{\underset{\infty }{\sum }}\left[\sigma_{t}^{F}\cdot V_{j}^{F}\cdot \left(1-\frac{\sigma_{t}^{F}\cdot d^{F}}{4\cdot \tau \cdot l_{j}^{F}}\right)\right]\right)+\sigma_{t}^{m^{*}}\cdot \left(1-V^{F}\right)$$

where *f_C_* is separated into the orientation factor (*χ*_1_) and the contributions of subcritical and supercritical fibers and interfacial shear strength (*τ*), $L_{c}$ is the critical length (Equation (3)), *l^F^* is the fiber length, and *d^F^* is the fiber diameter. This model assumes that the contribution of the fibers to the strength of the compound is divided into two factors (*X* and *Y*). The reinforcement fibers are divided into the largest and shortest fibers lengths that are considered critical (fibers with length less than $L_{c}$ are considered inefficient) [49].

(3) $$L_{c}=\frac{d^{F}\cdot \sigma_{t}^{F}}{2\tau }$$

The Bowyer–Bader solution to the Kelly–Tyson equation was applied. The Bowyer–Bader solution estimates the $\sigma_{t}^{F}$ as the product of the intrinsic tensile modulus of the fiber and the tensile elongation of the composite material ($\;\sigma_{t}^{F}=E_{t}^{F}\cdot \varepsilon_{t}^{C}$) [50]. The Kelly–Tyson equation can be expressed as the orientation factor ($\chi_{1}$) by the sum of the contributions of fibers of subcritical length (*X*) and supercritical length (*Y*), plus the contribution of the matrix (*Z*); Equation (4).

(4) $$\sigma_{t}^{C}=\chi_{1}\left(X+Y\right)+Z$$

**Figure 7:**
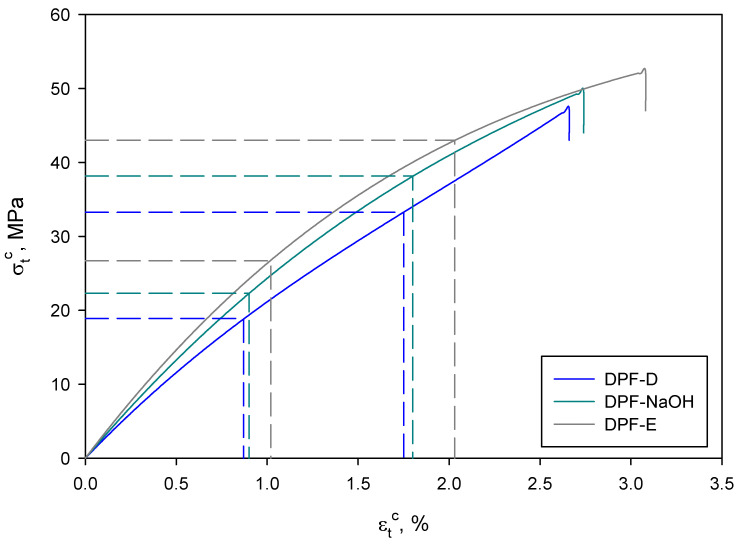
Elongation–tensile strength curves of the composites reinforced with 40% daste palm Figure 8. Fiber lengths distribution of 40% composites.

**Figure 8:**
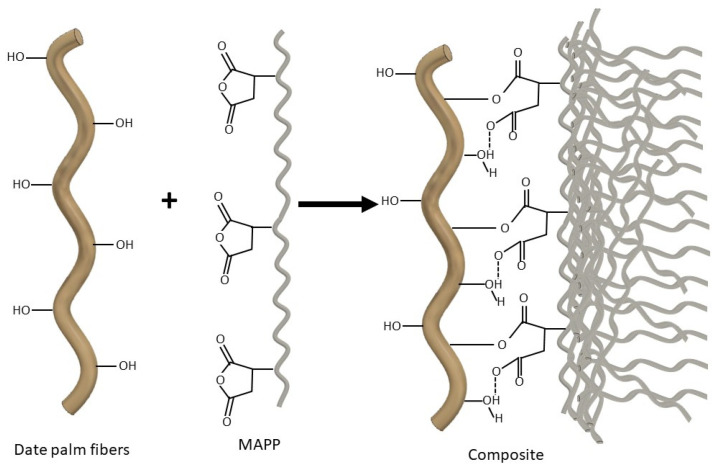
Scheme of date palm fibers interaction with MAPP.

Then, using two different strain levels and the maximum tensile strength at such strain levels (Figure 7) and length distributions (Figure 8 and Table 1), which were obtained by performing a morphological analysis of the fibers extracted from the composite materials, it is possible to estimate each contribution; (Equation (5)) [48,49].

(5) $$R=\frac{\sigma_{t1}^{C}-Z_{1}}{\sigma_{t2}^{C}-Z_{2}};\;R^{*}=\frac{\left(X_{1}+Y_{1}\right)}{\left(X_{2}+Y_{2}\right)}$$

As can be seen from Table 3, the results of the micromechanical analysis revealed a critical length of about 540 µm. However, in the case of enzyme-treated fibers, this critical length decreased to 412 µm. This may be due to the cutting action performed by the enzymes on their specific region for each enzyme. On the other hand, it can be observed how the value of the intrinsic tensile strength of the fiber increased for the treatments with sodium hydroxide and with enzymes. This increase in intrinsic strength is closely linked to the improved stress transfer. As mentioned above, the removal of lignin results in the availability of a greater number of hydroxyl groups on the fiber surface, improving their anchorage to the maleate polypropylene chain and the extent its reaction, which is in turn linked by an entanglement mechanism to the polypropylene chains. In addition, interface quality is influenced by the intrinsic strength of the fiber, the type of bond generated, and the number of bonds [36].

**Figure 9:**
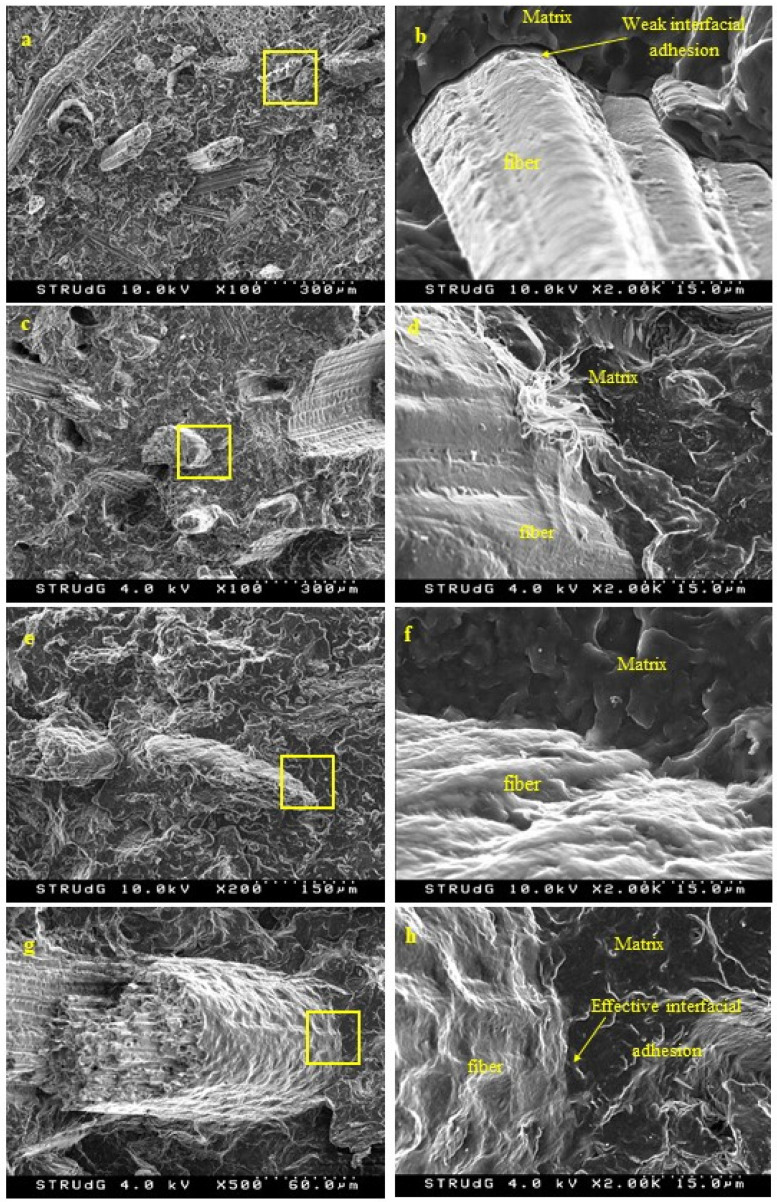
SEM micrograph of the tensile fractured surface for (**a**,**b**) DPF-D; (**c**,**d**) DPF-D-MAPP; (**e**,**f**) DPF-NaOH-MAPP; (**g**,**h**) DPF-R-MAPP.

On the other hand, the results obtained for the interfacial shear strength were close to the Von Mises criterion ($\tau =\sigma_{t}^{m}/\sqrt{3}=15.9$), and therefore slightly higher than those obtained by the Tresca one ($\tau =\sigma_{t}^{m}/2=13.8$) [51,52]. The increase in τ value, which corresponded to 15.01 for mechanically treated fibers and 15.70 for fibers with enzymatic treatment, corroborates the improvement in the interface due to the action of the enzyme on the lignin (Figure 9).

Lower lignin content in the surface chemical composition of the fibers leads to a greater number of hydroxyl groups. These superficial hydroxyl groups are available to interact with the coupling agent as detailed in Figure 8, improving the shear strength. This improved interface can also be observed by SEM (Figure 9). A comparison of the results obtained with the same composite using mechanical wood fibers shows that for a critical length of 577.6 μm and a similar intrinsic fiber strength (612.2 MPa), the value of interfacial shear strength is of the same order (15.95 versus 15.70) [18].

**Figure 10:**
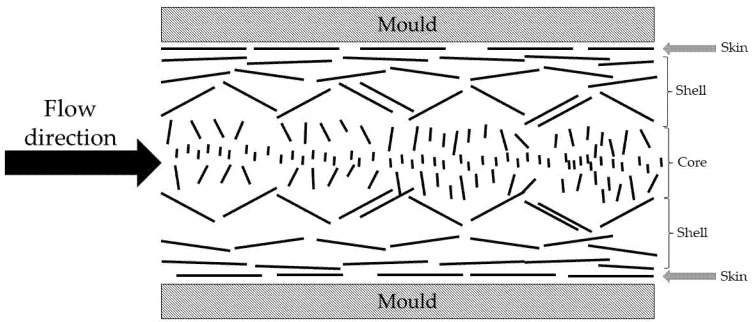
Scheme of fibers orientation inside the composite during injection molding.

In Figure 10, it can be seen that fractures occurred mostly across fibers, meaning that the stress is effectively transferred to fibers in the coupled composite, thus accounting for the superior tensile strength. The use of MAPP promoted the interaction between the natural fibers and the PP matrix at the interface, thus improving the fiber–matrix compatibility. As previously discussed, the proposed coupling mechanism is hydrogen bonding and covalent ester linkage generated by the chemical reaction of the anhydride groups of the MAPP and the hydroxyl groups of the fiber surface [53,54,55,56]. The efficiency of MAPP as a coupling agent in composites based on polyolefin matrix and natural fibers has been well established in the literature, and is even used on an industrial scale in WPC [18,19]. Owing to the presence of the maleic anhydride moiety grafted onto the PP backbone, MAPP is able to ensure a chemical continuity between lignocellulosic fibers and PP matrix by chemically binding the two phases through an ester linkage after condensation of the pendant MA with the surface hydroxyl groups of fibers. The addition of MAPP also improved the dispersion of lignocellulosic filler within the matrix by promoting the wetting of the filler with the hydrophobic polymer. One could therefore expect the DPF-NaOH fibers to be the reinforcement with the highest reinforcing potential, especially in the presence of MAPP. In fact, during the alkali treatment, part of the soluble lignin at the surface of the fibers was removed, exposing a higher amount of hydroxyl groups at the surface of the fibers [57]. However, Figure 10 shows that enzymatic treatment was the most appropriate treatment in terms of reinforcing potential. In fact, it has been shown that the surface chemical composition is crucial for the development of well-bonded composite materials.

The orientation factor is a measure of the average orientation of the reinforcement in the composite material relative to the direction of the tensile load. As mentioned above, this value is mainly affected by the geometry of the mold and the injection machine used. To determine the orientation factor, it should be considered that, generally, in injection-molded pieces, three regions are present: the skin layer, the shell layer, and the core layer. The skin layer appears at the border of the mold and consists of a thin layer of fibers oriented in the flow direction. Located directly under the skin, the shell layer is highly oriented in the direction of flow. In the central area, the fibers are oriented transversely to the flow (Figure 10) [56,58]. Therefore, the orientation factor represents the average of the alignment of the fibers in the flow direction. Consequently, the angle corresponds to the average angle of the different regions.

The obtained values, close to 0.33, are within the range established in previous works for the equipment (between 0.25 and 0.35) [51,59,60]. These orientation factor values can be translated to angles (α) by applying $\chi_{1}=cos^{4}\left(\alpha \right)$ [61]. From this we can obtain values for α in the three cases that are between 40.6 and 41.1. These values are similar to those obtained in previous studies [50].

Similarly, in the simulation of composite tensile strength with all of the fibers perfectly aligned in the direction of the test ($\chi_{1}=1$ in Equation (2)), the composite material would reach tensile strength values of 130.85 MPa for the enzymatically treated fibers.

The evolution of the interface resistance can also be evaluated by the value of the coupling factor ($f_{c}$). As mentioned above, the coupling factor comprises the fiber orientation factor and the length and interface factor ($f_{c}=\chi_{1}\cdot \chi_{2}$).

(6) $$\begin{array}{c} \tau =\frac{\sigma_{t}^{F}\cdot d^{F}}{4\cdot l^{F}\cdot \left(1-\chi_{2}\right)}\;when\;l^{F}>L_{c}\;\\ \tau =\frac{\sigma_{t}^{F}\cdot d^{F}}{l^{F}}\cdot \chi_{2}\;when\;l^{F}<L_{c} \end{array}$$

The results of the micromechanics show a decrease in the coupling factor, from 0.21 for DPF-D to 0.19 for DPF-E, as a consequence of the decrease of $\chi_{2}$. This decrease is a consequence of the reduction of the value of *l^F^*/*d^F^* due to the action of the enzyme. However, it is compensated by the better interface (Equation (6)) [47].

Finally, Table 4 compares the estimated results for the fibers’ tensile strength factors based on the equation of the rule of mixtures and the values obtained through micro-mechanical analysis.

As can be seen, an excellent correlation was found between the tensile strength factor of the fibers calculated based on the rule of mixtures and the values obtained based on the micro-mechanical analysis. This correlation confirms the results obtained, relating the increase in the reinforcement capacity of the fibers obtained by enzymatic treatment to the higher intrinsic properties of the fibers and an improved interface.

## 4. Conclusions

In this work, the processing of date palm residues to obtain high-yield fibers for use as PP reinforcement was studied. The work highlights the feasibility of using fibers from date palm residues as reinforcement in the production of composite materials, increasing the tensile strength of PP in the presence of MAPP by between 71 and 90%, depending on the type of DPF, when the reinforcement amount was set to 40 wt %. In addition, the Young’s modulus of the composite materials exhibited values significantly higher than the matrix, enhancing this property by between 161 and 205%. It was found that the morphology of the fibers, as well as their chemical compositions, play key roles in their reinforcing potential, especially due to their effects on the interfacial shear stress, and therefore on the interface between matrix and reinforcement. In addition, depending on the treatment, the intrinsic tensile strength of the fibers was also modified. In this sense, those fibers obtained by fully mechanical methods exhibited the lowest intrinsic tensile strength, while those enzymatically treated accounted for the highest.

Overall, the present work brings to light the potential valorization of date palm residues for the reinforcement of fiber-reinforced composites, giving a second life to this biomass and contributing to the environment by means of presumably more sustainable PP-based products.

## Figures and Tables

**Table 1:** Mechanical properties of 40% *w*/*w* Date palm fibers—PP composites.

Composite	V^F^	$\sigma_{t}^{C}$ (MPa)	$E_{t}^{C}$ (GPa)	$\varepsilon_{t}^{C}$ (%)	$\sigma_{t}^{m^{*}}$ (MPa)	$d^{F}$ (µm)	$l_{w,w}^{F}$ (µm)	lw,wFdF
PP	0	27.60 ± 0.13	1.50 ± 0.06	9.3 ± 0.2	27.6	-	-	-
PP-40DPF-D	0.315	47.17 ± 0.21	3.92 ± 0.04	2.65 ± 0.4	16.98	29.8	682	22.9
PP-40DPF-NaOH	0.307	49.55 ± 0.19	4.35 ± 0.09	2.73 ± 0.2	17.31	27.4	650	23.7
PP-40DPF-E	0.291	52.33 ± 0.17	4.57 ± 0.03	3.08 ± 0.5	18.63	18.0	435	24.2

**Table 2:** Chemical composition of the obtained DPF.

Reinforcement	Extractives (%)	Ash (%)	Lignin (%)	Holocellulose (%)	Kappa Number
DPF-D	2.59 ± 0.12	7.41 ± 0.16	26.50 ± 0.31	63.50	87.5
DPF-NaOH	1.98 ± 0.08	5.70 ± 0.24	24.63 ± 0.22	67.69	66.6
DPF-E	0.72 ± 0.07	1.97 ± 0.14	19.86 ± 0.17	77.45	43.9

**Table 3:** Main results of the micromechanical analysis.

Reinforcement	$L_{c}$ **(µm)**	$\sigma_{t}^{F}$ **(MPa)**	τ	$\chi_{1}$
DPF-D	537	541	15.01	0.323
DPF-NaOH	543	607	15.32	0.331
DPF-E	412	719	15.70	0.333

**Table 4:** FTSF compared to micromechanical results.

Reinforcement	FTSF	$f_{c}\cdot \sigma_{t}^{F}$
DPF-D	112.8	113.6
DPF-NaOH	122.3	121.4
DPF-E	134	136.6
